# Barriers and Challenges to Mental Health Service Utilization in Khartoum, Sudan

**DOI:** 10.1192/bjo.2025.10087

**Published:** 2025-06-20

**Authors:** Sara Elidressi, Rayan Ahmedalgabri, Radwa Ali, Hala Elhardlu

**Affiliations:** ^1^South London and Maudsley NHS Foundation Trust, London, United Kingdom; ^2^Sudan Medical Specialization Board, Khartoum, Sudana; ^3^University of Medical Sciences and Technology, Khartoum, Sudan; ^4^St Patrick’s University Hospital, Dublin, Ireland; ^5^Northamptonshire NHS Foundation Trust, Northampton, United Kingdom

## Abstract

**Aims:** Mental health disorders represent a significant burden globally, yet access to psychiatric care remains limited, especially in low- and middle-income countries. In Sudan, the utilization of mental health services is restricted by financial constraints, social stigma, and lack of service availability. This study aims to identify key barriers affecting mental health service utilization in Khartoum, assess the availability and affordability of essential psychotropic medications, and explore their influence on patient access to care.

**Methods:** A cross-sectional hospital-based study was conducted from October to December 2022 at Tigani El-mahi Psychiatric Teaching Hospital and Taha Bashar Psychiatric Hospitals in Khartoum. A stratified random sample of 384 psychiatric outpatients and their caregivers was interviewed using a structured questionnaire covering demographics, accessibility, affordability, stigma, and attitudes toward psychiatric care. Additionally, the availability of 24 essential psychotropic medications was assessed in public and private pharmacies. Ethical approval was obtained, and informed consent was secured from all participants.

**Results:** The most commonly reported barriers to mental health service utilization were financial constraints (34.4%), limited-service availability (21.4%), and stigma (10.9%). Over 84% of participants reported no psychiatric services within their locality, 49.5% travelled 1–3 hours, while 24.2% travelled more than 3 hours to access care. Medication shortages were significant, with the availability of essential psychiatric drugs ranging from 16.1–28.6% in public hospital pharmacies and hardly exceeding 37.5% in private pharmacies. Affordability was a major concern, with 70.3% of participants stating that prescribed medications were unaffordable and difficult to purchase. Education level was significantly associated with healthcare-seeking behaviour (p=0.018), with university-educated individuals more likely to seek treatment. These findings align with studies from other LMICs, where financial and accessibility challenges are similarly identified as major barriers to psychiatric service utilization.

**Conclusion:** Mental health service utilization in Sudan is severely impacted by financial constraints, limited-service availability, and stigma. Addressing these barriers requires integrating psychiatric care into primary healthcare, expanding community-based services, and ensuring the affordability and availability of essential psychotropic medications. Subsidized medication programmes, targeted community outreach, and mental health literacy initiatives could play a key role in improving accessibility. These findings contribute to the global discourse on mental health equity in resource-limited settings and underscore the urgent need for policy reforms and investment in mental health infrastructure.

**Funding Statement:** No external funding was received for this study.

